# Ingluvin

**Published:** 1886-12

**Authors:** 


### INGLUVIN.

A very learned name for a remedy is ingluvin. It is the essential principle of the gizzard, and bears the same relation to poultry that pepsin does to the higher animals. The honor of its discovery and utilization, in its crude state, lies remotely with the Chinese gastronomer, as well as to the Caucasian chemist, in its refined condition. From time immemorial the inhabitants of the Celestial Empire have used the gizzard of chickens and ducks in nearly all made dishes. The writers have recommended the practice as a sovereign treatment of dyspepsia, weak stomach and vomiting. A favorite prescription of Chinese physicians for chronic indigestion is to cut up and digest chicken gizzards in hot water until they are reduced to a pulp, and then add some spices. A tablespoonful or two of the resulting paste is taken at each meal until the patient has entirely recovered. From China the practice passed to other parts of Asia, and was adopted here and there among the Mediterranean people. Strange to say it was never learned by the great nations of Europe until the latter part of the present century. On the other hand, the organic chemists of Europe discovered,

1850, a powerful nitrogenous radical in the gizzard. Experiments thereafter showed it to possess many of the qualities of pepsin. These experiments lead to its isolation. Numberless experiments have proven it to be a very valuable addition to therapeutics. When pepsin refused to act, and where, in severe cases it has even been rejected by the stomach, ingluvin effected relief rapidly and with the greatest ease.

In four recent cases of poisoning by root beer, after pepsin and all similar compounds had been rejected by the stomach, ingluvin stayed the retching and enabled it to retain and digest food.

Our poultry are chiefly granivoaes, and have no beak nor other buccal apparatus for crushing the hard grains and seeds on which they so largely feed. The food is swallowed when apprehended and passes directly into the crop or gizzard. This seems to act both mechanically and chemically. Its interior walls are covered by a dense, hard cultous membrane, surrounded by muscles of the most powerful type. Along with the food is always a small amount of sand and gravel. The organ acts apparently by bruising and crackling, rather, than is commonly believed, by trituration. The motion of the ingluvial muscles is accompanied by a slow but continuous exudation from the walls of the crop of a strong organic fluid, of which ingluvin is the chief constituent. The hull of the grain or shell of the seed is broken by the pressure of the walls and the gravel, and their interior is exposed to the chemical action of the ingluvin. By the time it reaches the stomach it is ready for the gastric juices. From this point on, digestion proceeds as with the higher animals. As the gallinaceæ have very small salivary glands, and as the fluids secreted by these resemble the secretion of the parotid rather than that of the sublingual and submaxillary glands of the human being, it would seem as if ingluvin played a double part, exercising the function of the ptyalin of the saliva as well as the pepsin of stomach.

It has the remarkable property of arresting certain kinds of vomiting —notably the *vomiting of pregnancy*. It is a stomachic tonic, and relieves *indigestion*, *flatulence* and *dyspepsia*. It can be administered in inflammatory conditions of the mucous membrane, as it has no irritant effect. Under ordinary circumstances, and when the object of its administration is to promote the digestive function, it should be administered after meals. When the object is to arrest the vomiting of pregnancy, it should be given before meals.—*American Analyst.*

